# Abstracts from German Journals

**Published:** 1873-03

**Authors:** Ch. Rauschenberg

**Affiliations:** Atlanta, Ga.


### ABSTRACTS FROM GERMAN JOURNALS.

BY CH. RAUSCHENBERG, M.D., ATLANTA, GA.

### *ON THE CATARRH OF THE GENITAL ORGANS OF THE FEMALE— (FLUOR ALBUS).*

The views on the pathology and treatment of this most common disease of females, as they are contained in the following pages, are those of Professor Hildebrandt, of Königsberg, Germany. A clinical lecture on the subject, delivered by this very able gynæcologist before his class, has furnished the material, and has been used for the basis of this paper, like others from the same work* in former numbers of this journal, for the purpose of bringing the conceptions of German authorities, and the modifications which the views of others have undergone there, to the consideration of American practitioners, and of incorporating them into the medical literature of this country.

Fluor albus, a frequently copious and always debilitating discharge from the genital organs of the female, is not, as it is but too frequently considered, a secretion from the vagina, but has its origin much oftener in the uterus and its appendages, or at the very commencement of the sexual canal, the vestibulum vaginæ, then in the vagina itself.

The microscopical and chemical examination of this secretion first, and a careful local inspection of the sexual organs next, will confirm this fact, and enable the physician to determine the locality of the genital canal, whose mucous membrane gives origin to this morbid secretion.

*Volkmann's Sammlung, Klinischer Vorträge.

Besides the marked differences which this discharge exhibits in relation to color, consistency and odor—having sometimes the appearance of a thin turbid serum, or of a thick, creamy, or blood-colored pus, or of a tenacious, glassy albuminous mucous, or again of a dirty gray, or brownish, more or less fœtid lochial ichor—it shows under the microscope in different cases not only prominently pavement epithelium, infusoria (trichomonas vulvæ) and fungi (leptothrix vaginalis, oidium albicans), as they occur in the vagina alone, but more frequently and abundantly globules of mucus, cylinder and fimbriated epithelium.

The thin, turbid serum, the pavement epithelium, the infusoria and fungi constitute the microscopical elements of vaginal; the albuminous mucus, with its numerous mucous globules and cylinder epithelium those of the cervical; and the fimbriated epithelium those of the uterine catarrh; and as one or the other of them prevails prominently under the microscope, it is proper to look for the seat of the disease principally in the locality which they represent. The chemical test of the leucorrhœal discharge is more difficult and less reliable, as the secretions from the different portions of the canal are necessarily more or less mixed. The secretion of the mucous coat of the corpus uteri, obtained by the aid of the uterine syringe, is generally found of neutral, that of the cervix of strong alkaline, and that of the vagina of sour reaction. As the one or the other predominates in the discharge, we are justifiable in suspecting the part of the canal, which it represents, as prominently affected.

Ocular inspection of the genital organs, with the aid of the speculum, furnishes the most reliable diagnosis. Dr. Hildebrandt uses for this purpose, in a majority of instances, the cylindrical glass speculum of Ferguson, as the most convenient, most applicable and efficient instrument; unless morbid conditions of the vagina or displacements of the uterus prevent observations of the cervix with this instrument, Sims' speculum, and still oftener Cuscos' bivalve instrument, as it requires no assistance like Sims', are resorted to.

The chronic catarrh of the genital mucous membrane, particularly of the vaginal portion of the uterus, being of much more frequent occurrence than the acute, and that of other portions of it, our author first enters into a consideration of the phenomena presented by that form of the disease.

The image of cervical catarrh, as presented by the speculum, in virgins and females who have never borne children, as well as the morbid consequences resulting from it in these two classes of patients, are essentially different.

In the first class, the cervix is abnormally elongated, thickened above, sharp and pointed below, of a dark red, or bluish red color, with circular abrasion of the epithelium, or ulceration around the os, from which frequently a drop of tenacious, glassy, more or less purulent secretion protrudes. In the second, the cervix generally has a cylindrical form, is generally broader across the elongated vertical orifice than it is higher up; also of a deep red color, and the broad, swollen lips of the os are, in old cases, generally covered with erosions. On touching the latter, or enteriug the generally dilated cervical canal with the finger, the swollen mucous membrane exhibits a velvety softness, and frequently hypertrophied cervical follicles and papillæ are felt, as more or less round, granular elevations. The characteristic features of cervical catarrh, in both classes of females, thus consist in *thickening of the mucous membrane with secondary participation and enlargement of the entire cervix, increase and thickening of the glassy secretion, desquamation of the epithelium, and hypertrophic enlargement of the denuded glands and papillæ.*

The necessary local and constitutional consequences of this disease, although it affects only a very small portion of an organ not very sensitive within itself, are generally very troublesome, and sometimes dangerous.

In virgins, and females who have never borne children, a cervical catarrh of longer duration induces always abnormal local hyperæmia, which causes frequently excessive, sometimes too frequent, and occasionally, but more seldom, painful menstruation. Ulcerations, where such already exist, give rise to intercurrent uterine hemorrhages, in consequence of friction of the ulcerated os against the numerous and rigid transverse rugæ of the posterior walls of the vulva during active motions of the body. Cicatrices caused by the healing of such ulcerations establish contraction of the external os, the abnormally thickened and tenaciously adhering secretion of the cervical follicles stagnates, obstructs the cervical canal, and accumulates within the cavity of the corpus uteri. At this period, the entire womb presents itself, enlarged in length and breadth, as a thin-walled soft balloon, from which the morbid secretions of the cervix, as well as those of its own mucous membrane, which generally becomes secondarily involved, are occasionally expelled by painful, labor-like contractions of the organ in the form of a yellowish, purulent, more or less thickened fluid. Pressure of the intestines from above is no longer resisted by the enlarged and flabby uterus, it leaves its normal position, and gives way posteriorly in the direction of Douglas' space, and thus retroflection becomes one of the most frequent secondary consequences of the cervical catarrh of virgins.

Sterility, even if marriage is entered into in the early stages of the disease, is almost without exception the lot of such females. Thickening of the mucous membrane, with its close-fitting palmæ plicatæ, hypersecretion, thickening and stagnation of the same within the cervical canal, and subsequent posterior flexion of the attenuated and expanded corpus uteri, establish indeed three very substantial obstacles to the entrance of sperm into the uterine cavity.

Among the secondary consequences, nervous irritation of the stomach, similar to those of pregnancy—to wit, gastrodynia, want of appetite, with occasional harrassing sensations of hunger (bulimia) which, on account of the irritability of the stomach, can not be appeased, and now and then vomiting—occur first; irritation of the bladder, with frequent inclination to pass urine, next, and neuralgic pains in the dorsal, or the lumbar and inguinal regions, extending along the thighs, from mechanical irritation of the ulcerated os, or displacement of the body of the womb, or both together, restrict active motions, and cause the patient to seek the couch even frequently during the day. Thus, deficient digestion and nutrition, disturbed sleep, general nervous irritation, and want of exercise in the open air, undermine the health of the patient, and an apparently trivial affection of that small portion of the womb, which physiologically has no essential connection with the principal functions of that organ —to wit, menstruation and gestation—becomes the fruitful source of a large number of the cases of hysteria and chlorosis, which come so frequently under the treatment of practitioners, and which but too often, at least as far as chlorosis is concerned, are erroneously considered the cause instead of the consequence of the cervical catarrh.

While in virgins, or females who have never given birth, the secondary participation of the cervix, the destruction of the faculty of conception and the occurrence of irritative phenomena in different distant spheres of the nervous system, constitute the principal dangers connected with cervical catarrh; the fact that in females who have by repeated births had their cervix extended and converted into a funnel-shaped canal, with the largest diameter at the external os, from which all secretions can readily flow, changes the phenomena and the consequences of the disease very materially in that class of females. The cervical canal remains in these cases entirely unobstructed, hence the faculty of conception is not interferred with, the position of the organ is not changed, and the general health not affected in the same manner as in virgins, unless, as it happens in a few rare cases, the copiousness of the albuminous secretion causes too much loss of substance, and consequently emaciation. Extensive degeneration of the mucous membrane, oftenest on the lips of the os, and less frequent at other portions of the cervical canal as far as the internal os, are in this class of females the prominent sources of subsequent trouble. Long lasting cervical catarrhs in females who have given birth to several children, lead almost in every case to ulceration of one or both lips. The anterior one, over which the alkaline secretion of the cervix has been permanently flowing since the existence of the disease, is generally most prominently affected. Where the ulceration continues unarrested for a length of time, it generally spreads gradually and converts the entire anterior lip of the cervix into one suppurating surface, whose secretion mixes with the glassy mucus of the cervical canal. Sooner or later small elevations and excrescences, representing different pathological formations, make their appearance on the surface.

In one group of cases, these excrescences are formed by hypertrophic enlargement of the glands, imbedded within the mucous membrane. They are of different size, enclose cyst-like cavities, the enlarged gland-tubules, and develop themselves finally into those dark red, soft tumors, with long pedicles, which are usually called mucous polypi, and occur along the entire length of the cervical canal often in great abundance. Some of them attain the size of a cherry, while the most of them are smaller.

In another group of cases it is the connective tissue between the glands, which grows and elevates itself into small, circumscribed plaques above the level of the mucous membrane. Small cavities, filled with a serous fluid, are developed, and thus tumors of different size, sometimes with broad adhesions, sometimes pediculated, make their appearance, which are also frequently called mucous polypi.

These two forms of growths, occurring on the soft, spongy, hypertrophied cervix of females suffering with cervical catarrh, become principally injurious to the general health by the profuse effusions of serous fluid and the copious menstrual or irregular uterine hemorrhages which they invariably occasion, most frequently in consequence of the every-day mechanical irritations or occasional coarser insults of the diseased neck.

Much more serious consequences follow a third form of pathological growths, connected frequently with cervical catarrh—to wit: those which have their origin in the papillæ of the mucous membrane. Females with a hard, unyielding and rather small vaginal portion of the uterus, are particularly subject to them. Where the uterine orifice is wide and open, small elevations are seen on the ulcerated patches, which bleed freely when touched; later they ramify with each other, swell and extend into bulbous elongations. This process goes on quicker on the lips than within the cervical canal. Where the orifice of the womb is narrow or closed, they protrude from it as flattened excrescences, similar in appearance to the comb of a rooster—the cauliflower excrescences of our writers. They are of very serious prognosis, as, according to Prof. Hildebrandt's experience, far over one-half of all the cancerous degenerations of the vaginal portion of the womb originate in cervical catarrh with papillary vegetations.

Amongst the causes giving rise to cervical catarrh, our author considers gestation and delivery, even as they normally occur, by far the most common instigators of the disease. The generally hyperæmic mucous membrane of the enlarged and softened cervix uteri, in the last month of pregnancy, with its thick, cream-like secretion in consequence of increased exuviation of epithelial cells, accounts for the erosions of the epithelium about the lips of the womb so often observed at that time. These, at this period, almost normal, shallow ulcerations of the cervix, heal very soon after quick and easy deliveries; but where, in cases of rigid os, dry or otherwise tedious labor, the head of the fœtus presses the denuded and attenuated lips of the uterus, perhaps for hours, against the anterior walls of the pelvis, or causes them to become deeply lacerated, or where, perhaps in addition to all this, the female gets on her feet before these injuries have been repaired by nature, or neglects the necessary cleanliness, or suffers in consequence of imperfect delivery of placenta or decidua, etc., from an ichorous and fœtid discharge, an increase of the ante-partum ulcerations in breadth and depth must necessarily follow, and a cervical catarrh must become permanently established.

Outside of pregnancy, delivery and puerperium, many different influences may produce cervical catarrh. Mechanical and chemical irritants and constitutional diseases, and amongst them masturbation, permanent labor on sewing machines, scrofulosis and tuberculosis, respectively, occupy the first rank.

The effects of masturbation, by the mechanical injury thereby inflicted, are necessarily catarrh, desquamation of the epithelium, excessive irritation and hyperæmia of the uterine and spinal nerves, relaxation of the muscular walls of the uterus and vagina, and hypersecretion, in which train of symptoms the cervix predominantly participates, in the same manner in which relaxation of the scrotum in males is accompanied by hypersecretion of the prostate gland and testicles.

The use of sewing machines, particularly in already debilitated females, produces by friction of the labia against each other, very similar phenomena. On examination, sometimes after they have only been used a few weeks, the vestibulum and the introitus vaginæ are discovered abnormally red, and the latter covered with a copious secretion, the cervix also swollen, very tender, and sometimes, when the cause has been in operation a longer time, ulcerated. The females look generally pale, complain of weakness, headache, vertigo, and suffer from general nervous irritability. That these symptoms are not alone owing to the noise of the machine and the tension in which the nervous system is kept by the close attention and prompt manipulations which the changes of the position of the material under operation require, has been proven to our author by the frequent observation of the fact that sewing machines worked by hand produce none of these effects.

The experience of many years has convinced Professor Hildebrandt that general constitutional diseases can find their localization in the mucous membrane of the vaginal portion of the uterus: He has often seen young girls with intense cervical catarrh, glassy discharge, bulbous enlargement, and great tenderness of the cervix, normal uterus and healthy vagina, where no mechanical causes, no previous acute imflammation of the uterus, could be established; but where the insidious and slow development of the disease, the fair condition of the general health, indurated cervical glands, characteristic cicatrices on the neck, nasal catarrh, and the established fact that they had been excessively scrofulous during childhood, left no doubt that the process of scrofulosis had not entirely run its course.

The large number of cases, and the indubitable etiological facts established in connection with them, leave no doubt on his mind that the mucous membrane of the cervix uteri is just as much a center of localization of scrofulosis as that of the nose, the eyes, the ears, etc.

He has also frequently observed the disease in married tuberculous females, but has not been able to decide how much tuberculosis has to do with its origin, as the number of unmarried females afflicted in the same manner, which have come under his observation, has not been sufficiently large to enable him to arrive at definite conclusions. No form of catarrh of the uterus is, however, in his opinion, so obstinate, and resists local treatment so long, as that occurring in tuberculous females.

He does not agree with Scanzoni, in relation to chlorosis as a cause of cervical catarrh, on the supposition of that writer that the inability of the weak and flabby heart in that disease, to supply the uterus and sexual organs with a sufficient quantity of blood to keep up regular and normal menstruation substitutes for it a low, chronic hyperæmia of the sexual organs, chronic engorgement of the uterus, enlargement of the organ, and hypersecretion of its membranes.

He considers that, on close scrutiny, the causes of cervical catarrh connected with chlorosis will be found various and often—such ones as do not depend upon the condition of the blood, or the circulation of the chlorotic patient.

In many females, chlorosis is, beyond a doubt, the consequence of masturbation; in others, it is developed upon the basis of infantile scrofulosis, or hereditary tuberculosis, and then the cervical catarrh can easily be recognized as the result of the same cause which produced the chlorosis itself.

Where chlorosis existed, as a primary disease of the blood *sui generis*, our author has always discovered, behind a narrow but generally relaxed hymen, a loose-walled vagina, and an unusually movable, soft and relaxed uterus. Catarrhal symptoms were found, in a large majority of cases, *only* in the vagina, very seldom in the corpus uteri, and also quite seldom in the cervix; and he considers their occurrence the result of the mechanical friction connected with the motions of the body, and the pressure of the bladder increased by the general relaxation and collapse of the sexual organs in that disease.

The nearest pathological relationship to the cervical catarrh, from constitutional causes, bears that particular catarrhal affection of the genitals which has been called by our author vaginitis exulcerans adhesiva. It occurs principally in females disposed to arthritis and diseases of the liver after the period of fecundity has been passed, with troublesome general phenomena, such as nausea, irritations, want of appetite, costiveness, fœtid urine, with abundance of lateritious sediment, excessive nervousness, and a copious, acrid fluor albus, causing excoriations on the labia and inner surface of the thighs. The speculum reveals a very moist vagina, reddened moderately about the introitus and upwards for two-thirds of its depth, while the upper third, particularly immediately around the cervix, and the cervix itself, are of a dark red color, excessively tender, entirely destitute of epithelium, and covered with a sticky, sanguino-purulent secretion. If this condition exists any length of time, agglutinations of the vagina and cervix, complete adhesion of the two, and disappearance of the cervical portion, are the consequences. The corpus uteri generally remains uninvolved until a very late period of the existence of the disease.

In summing up the anatomical reasons for the occurrence of this long train of morbid conditions of the small cervix uteri, physiologically the most unimportant part of the womb,

Professor Hildebrandt concludes that the prominently passive position which it occupies during the process of labor, the severe tension, contusions and lacerations which it frequently undergoes during that time, its comparatively smaller power of contractility to resume its normal position and size afterwards, simultaneously with the much more muscular body of the womb, its position within the vagina exposing it much more to direct injuries, and then again the anatomical structure of its mucous membrane with its abundant and close-fitting folds, its rich supply of easily hypertrophied glands and papillæ, its monthly congestion during the period of menstruation, and withal the narrowness of the canal, are abundantly sufficient to account as well for the frequency as the obstinacy of this disease.

As neither the anatomical position, nor the structure of the mucous membrane of the corpus uteri, offers alike favorable conditions for the development of that pathological process, catarrhal affections of that portion of the womb are comparatively much rarer. When they exist they establish less characteristic symptoms, and are harder to diagnose. A watery, thin, mostly very copious dscharge, without local trouble otherwise, is the most prominent symptom. The patient feels weak, nervous, looks pale, and sooner or later suffers with gastric disturbances; the menstruation is often normal, at the commencement of the disease often too copious, after a long duration of the same generally scant. The faculty of conception is gradually lost, most probably in consequence of a change of the fimbriated epithelial cells into cylinder and afterward into polymorphous cells. Hence, sterility and a copious thin secretion remain as the two principal symptoms of this disease.

Whether this copious secretion has its origin from the cavity of the womb, or its neck, neither the speculum nor the touching finger can determine. The above-mentioned microscopical and chemical tests are furnished, but perfect certainty in relation to this question can only be attained if, on trial with a uterine injection syringe, a quantity of fluid can be withdrawn from the uterine cavity. It can be done in all cases where catarrh of that cavity has become well developed, and the very existence of the fluid within that cavity establishes the diagnosis. The yellowish-red color, the great liquidity of the fluid, and the presence of fimbriated epithelium, are additional confirmations of its correctness. In old cases, and this is not an unimportant prognostic criterion, the fimbriated epithelium is often lacking, and cylinder or polymorphous epithelium cells take its place.

In relation to the causes of the intra-uterine catarrh, it is, in many cases, impossible to arrive at definite conclusions. Our author thinks himself justified by the experience of many years to look upon too long continued lactation, particularly when births follow each other in quick succession, in delicate and weakly females, as the most common cause of the disease. Irritation of the spinal cord, relaxation of the integuments of the uterus, and hypersecretion, occur in these cases, in the same manner as masturbation, only by the instrumentality of other nerve tracts.

In other cases, these cases are the simple consequence of obstructions of the internal os, occurring in consequence of flexions, senile atrophy, and internal cicatrices in cervical catarrh. Other cases again, originate during the puerperal state, in consequence of deficient retrogression of the body of the womb to it normal size, and deficient formation of new mucous membrane. Retention of the decidua after abortions, morbid growths within or beneath the mucous membrane, sarcoma, polypi, and small, circumscribed hypertrophic spots of the mucous membrane, which can not be diagnosticated in life, but show themselves as small, bluish red, bulbous elongations after death, are occasionally the causes of this disease.

Pathological anatomy has established the fact that intra-uterine catarrh frequently extends along the mucous membrane of the fallopian tubes. A diagnosis of that occurrence is impossible; even if cystic enlargement of these tubes (hydrosalpinx), in consequence of that catarrh, offers a palpable tumor for examination, it can not be distinguished from an ovarian cyst, or a parametritic exudation.

TO BE CONTINUED.

